# Supplementary material for: Context-dependent reversal of odorant preference is driven by inversion of the response in a single sensory neuron type
Source: PLoS Biol. 2022 Jun 13;20(6):e3001677. doi: 10.1371/journal.pbio.3001677 (PMC9232122; doi:10.1371/journal.pbio.3001677)
Supplement: S1 Table — (DOCX) [file pbio.3001677.s008.docx]

**S1 Table.** Strains used in this work.

| **Strain** | **Genotype** |
| --- | --- |
| WT | N2 (Bristol) |
| PY7502 | *oyIs85[ceh-36Δ*p*::TU813(recCaspase), ceh-36Δ*p*::TU814(recCaspase), unc-122*p*::dsRed, srtx-1*p*::gfp]* |
| PY12217 | *oyEx677[odr-1*p*::unc-103(gf)::SL2::mCherry, unc-122*p*::gfp]* Line 1 |
| JN1713 | *peIs1713[sra-6*p*::mCasp1, unc-122*p*::mCherry]* |
| PY10515 | *oyIs85[ceh-36Δ*p*::TU813(recCaspase), ceh-36Δ*p*::TU814(recCaspase), unc-122*p*::dsRed, srtx-1*p*::gfp]; peIs1713[sra-6*p*::mCasp1, unc-122*p*::mCherry]* Line 1 |
| PY12005 | *kyIs602[sra-6*p*::GCaMP3, unc-122*p*::gfp]* |
| PY10511 | *unc-13(e51); kyIs602[sra-6*p*::GCaMP3, unc-122*p*::gfp]* Line 1 |
| PY10513 | *unc-31(e928); kyIs602[sra-6*p*::GCaMP3, unc-122*p*::gfp]* Line 1 |
| PY10501 | *oyIs91[odr-1*p*::GCaMP3, srsx-3*p*::mScarlet, unc-122*p*::dsRed]* |
| PY10505 | *unc-31(e928); oyIs91[odr-1*p*::GCaMP3, srsx-3*p*::mScarlet, unc-122*p*::dsRed]* Line 1 |
| PY10507 | *unc-13(e51); oyIs91[odr-1*p*::GCaMP3, srsx-3*p*::mScarlet, unc-122*p*::dsRed]* Line 1 |
| CX2065 | *odr-1(n1936)* |
| PY10510 | *odr-1(n1936); oyIs91[odr-1*p*::GCaMP3, srsx-3*p*::mScarlet, unc-122*p*::dsRed]* |
| CX3222 | *odr-3(n1605)* |
| NL334 | *gpa-2(pk16)* |
| NL335 | *gpa-3(pk35)* |
| NL2330 | *gpa-13(pk1270)* |
| GJ006 | *gpa-2(pk16) gpa-3(pk35) gpa-13 (pk1270)* |
| GJ041 | *gpa-3(pk35) gpa-13(pk1270) odr-3(n1605)* |
| PY10520 | *odr-3(n1605); oyEx680[odr-1*p*::odr-3::SL2::mCherry, unc-122*p*::gfp)]* Line 3 |
| PY10509 | *odr-3(n1605); oyIs91[odr-1*p*::GCaMP3, srsx-3*p*::mScarlet, unc-122*p*::dsRed]* |
| PY10522 | *tax-4(p678); kyIs602[sra-6*p*::GCaMP3, unc-122*p*::gfp]* |
| PY10518 | *tax-4(p678); oyIs91[odr-1*p*::GCaMP3, srsx-3*p*::mScarlet, unc-122*p*::dsRed]* |
| PY10525 | *daf-11(m47); oyEx685 [odr-1*p*::GCaMP3, unc-122*p*::mCherry]* Line 1 |
| PY10526 | *daf-11(m47); oyEx686 [odr-1*p*::GCaMP3, unc-122*p*::mCherry]* Line 2 |
