# Supplementary material for: Context-dependent reversal of odorant preference is driven by inversion of the response in a single sensory neuron type
Source: PLoS Biol. 2022 Jun 13;20(6):e3001677. doi: 10.1371/journal.pbio.3001677 (PMC9232122; doi:10.1371/journal.pbio.3001677)
Supplement: Attachment — Submitted filename: Responses to Reviews_v5_Refs.docx [file pbio.3001677.s009.docx]

**Responses to Reviews**

Reviewer 1:

*1a. Calcium imaging is quantified by (F-F0)/F0. Formally, the reported changes could be due to F or F0. Normally it is assumed that F0 is constant, and changes are due to F. However, odorant saturation would be expected to change F0. This would affect comparisons between the presence and absence of saturation.*

*I agree with reporting the results as (F-F0)/F0 because it is most relevant to show on the changes from the F0 baseline since it reveals whether the odorant is changing the activity of the neuron.*

*Nonetheless, there might be conditions where a flip between increased and decreased (F-F0)/F0 are due to changes in F0 when during IAA saturation, rather than reflecting the total activity of the neuron. For example, if the drop in F0 caused by the presence of IAA dominates, then (F-F0)/F0 could flip from negative to positive.*

*This issue could be addressed by examining the F0 values with and without IAA saturation within existing data, which could help refine the interpretations of the calcium imaging without the need to add any additional experiments.*

The reviewer raises a valid concern. To address this issue, we have now included all relevant raw traces of the fluorescence changes in AWC in wild-type, *odr-1* and *odr-3* mutants in response to hexanol in control and sIAA conditions (new Figure S2C and Figure S5C). These traces not only show the F0 values but also show the magnitude and sign of the responses. While F0 values are lower in sIAA in wild-type (but not in *odr-1* or *odr-3* animals; see Figure R1 below), addition of hexanol clearly results in a large positive fluorescence change. These data indicate that the positive (F-F0)/F0 values reported are not simply due to a change in F0 in saturation conditions but reflect a change in neuronal activity.

*1b. Also, if there is a prior literature on whether the targets of AWC relevant to chemotaxis sense change or absolute levels, the information would also help resolve the issue.*

A general principle in *C. elegans* chemotaxis is that neurons respond to the change in concentration of the chemical as opposed to the absolute concentration. This principle enables *C. elegans* to robustly climb up or down chemical gradients spanning a range of concentrations by altering the probability of executing specific navigational strategies (Albrecht and Bargmann, 2011; Kunitomo et al., 2013; Larsch et al., 2015; Levy and Bargmann, 2020; Luo et al., 2014; Pierce-Shimomura et al., 1999). In the case of AWC, it has been shown that AWC sensory responses continuously adapt to changing odor concentrations as animals climb a gradient, and respond only when the concentration of the odor input is higher than the adaptation threshold (Levy and Bargmann, 2020). These responses of AWC to odor concentration changes are robustly translated into changes in synaptic signaling from AWC, and corresponding changes in intracellular calcium levels in the AIA, AIB, and AIY postsynaptic partners of AWC (Chalasani et al., 2010; Cho et al., 2016; Ventimiglia and Bargmann, 2017).

*2. It is counterintuitive that loss of odr-1 leads to increased calcium, since odr-1 is predicted to activate TAX-2/TAX-4. Is it because odr-1 mutants have a lower baseline calcium (related 1 above)? I suggest that the authors address this in the discussion.*

As shown in Figure R1 below, *odr-1* mutants do not have lower baseline calcium levels than wild-type in sIAA. Moreover, as noted above, addition of hexanol in sIAA conditions results in a large positive change in fluorescence in *odr-1* mutants (new Figure S5C), indicating that hexanol robustly increases calcium in *odr-1* mutants in sIAA.

In the original manuscript, we suggested that in sIAA, hexanol signal transduction occurs via an ODR-1-independent mechanism to increase intracellular cGMP and calcium levels. This may occur in part due to ODR-3 Gα protein-mediated activation of additional guanylyl cyclase(s) or inhibition of one or more phosphodiesterases. AWC is predicted to express at least six receptor guanylyl cyclases, as well as five phosphodiesterases (Taylor et al., 2021). The contributions of phosphodiesterases to mediating AWC olfactory responses are largely unknown. Of the AWC-expressed receptor guanylyl cyclases, in addition to ODR-1, the DAF-11 receptor guanylyl cyclase has been shown to modulate AWC olfactory responses (Birnby et al., 2000; Shidara et al., 2017).

**Fig. R1.** F0 values calculated from data shown in Figures 2A and S5B. Each dot is the F0 value from a single AWC neuron in response to hexanol in control or sIAA conditions. Alleles used were *odr-1(n1936)* and *odr-3(n2150)*. Long horizontal bars indicate the mean; errors are SEM. * indicates different at *P*<0.05 (Kruskal-Wallis test together withand posthoc pairwise Wilcoxon and Benjamini-Hochberg corrections).

We have now tested whether hexanol acts via DAF-11 to activate AWC in sIAA. As shown in new Figure S5D, we now find that hexanol-mediated activation of AWC in sIAA is partly affected in *daf-11* mutants. Our results suggest that ODR-3 may activate DAF-11 and additional guanylyl cyclases other than ODR-1, and/or inhibit one or more phospodiesterases, to increase intracellular cGMP levels in sIAA. The revised model is now described in the Discussion and presented in Figure 4D.

*3a. Page 6: "We infer that distinct underlying antagonistic neuronal pathways mediate attraction to, and avoidance of, hexanol at all concentrations. Attraction predominates at lower, and avoidance at higher, hexanol concentrations; sIAA inhibits the attraction but not the avoidance pathway.” The line of argument in this section requires some clarification.  Is this setting up the hypothesis that the mechanism for switching to avoidance for high hexanol is the same as for switching to hexanol avoidance under sIAA?*

That was not our intention. We only wished to make the point that antagonistic attraction/avoidance pathways operate to drive responses to hexanol at multiple concentrations, and that attraction predominates at low, and avoidance at high hexanol concentrations. By inverting the attraction-driving pathway to avoidance, sIAA shifts the curve towards avoidance at all concentrations. Since we have not studied the neuronal mechanisms required for avoidance of high hexanol concentrations here, we do not currently know whether or not additional neurons are implicated (as is often the case at high odorant concentrations). We have rephrased the relevant sentences in the Results section and also state that “Additional mechanisms may operate to drive avoidance of high concentrations of hexanol in control or sIAA conditions” in the Discussion.

*3b. It also appears that the authors are suggesting that the avoidance mechanisms for octanol, high hexanol, and hexanol under sIAA are the same, and trying to provide a single unified mechanism for three subtly different behaviors. I suggest that the discussion is a better place for this, since it does not really set up the precise question that authors address in subsequent sections.*

It was not our intention to compare the mechanisms that drive avoidance of octanol, high hexanol concentrations, and low hexanol concentrations in sIAA. We focus solely on the mechanisms that switch the response to low concentrations of hexanol from attraction to avoidance in sIAA, and in this work, have not studied the neuronal mechanisms required for avoidance of octanol or high hexanol concentrations which may be different. We have added sentences to the Discussion to clarify this.

*4. page 9: "...in unc-13 and unc-31 mutants that lack synaptic and peptidergic transmission, respectively (Sieburth et al., 2007; Speese et al., 2007)."This statement is inaccurate and should be revised. Sieburth 2007 shows that unc-13 has a peptidergic secretion defect that is just as strong as unc-31. Sieburth 2007 states in the abstract: "Similar neuropeptide secretion defects were found in mutants lacking unc-31 (encoding the protein CAPS) or unc-13 (encoding Munc13)."*

The reviewer is correct regarding the conclusions of the Sieburth *et al* 2007 paper (Sieburth et al., 2007). However, the effects of mutations in *unc-13* on peptidergic transmission are allele-specific as reported in (Speese et al., 2007). Thus while both papers showed that *unc-13(s69)* mutants exhibit defects in both synaptic and peptidergic exocytosis, Speese *et al* went on to show that *unc-13(e51)* mutants only exhibit synaptic but not peptidergic exocytosis defects (Speese et al., 2007). We used *unc-13(e51)* in all our analyses. We have clarified the use of specific alleles in the main text and also ensured that we describe the alleles used in the corresponding Figure Legends.

*5. The use of unc-13 and unc-31 to test cell-autonomy are good experiments, but these mutations do not affect signalling via gap junctions. The potential for gap junctions to play a role should be stated as a caveat.*

Done.

*4. In the section on testing the role of ASH, it seems to be implied that tax-4 affects AWC but not ASH. This should be clarified by stating whether tax-4 is expressed in ASH.*

*tax-4* is not expressed in ASH. We have added a sentence stating this.

*5. The figure or figure legends for 1G should indicate the genotypes of the AWC- and ASH- animals. Presumably these are the caspase strains listed in Table S1? Or were these laser-ablated?*

We have added a sentence to Figure 1D legend to indicate that the neurons were genetically ablated via the expression of caspases. We have also added this information to the relevant sections of the main text and we now refer the reader to Table S1 that describes the genotypes of these neuron-ablated strains.

*6. Different statistical tests and corrections for multiple hypotheses testing were used for similar data types. In the chemotaxis assays, Mann Whitney Wilcoxon or Kruskal-Wallis with posthoc pairwise Wilcox and Benjamin-Hochberg corrections were used for one set, and the two-way ANOVA with Bonferroni for another. The rationale for using different tests instead of 1 consistent test should be justified in the methods. If not, please use the same test for similar data* types.

In this manuscript, we used the Mann Whitney Wilcoxon, Kruskal-Wallis with posthoc pairwise Wilcoxon test and Benjamini-Hochberg correction, and ANOVA statistical tests to analyze behavioral and calcium imaging data.

*Calcium imaging:* All statistical analyses comparing only 2 groups were performed using the Mann Whitney Wilcoxon test. In analyses where the response amplitudes of 2 or more groups were compared, we used the Kruskal-Wallis test with posthoc pairwise Wilcoxon and Benjamini-Hochberg corrections.

*Chemotaxis behaviors*: We used two distinct types of behavioral assays in this work.

a) Plate chemotaxis assays: The Mann Whitney Wilcoxon test was used for all analyses where 2 groups were compared, and the Kruskal-Wallis test with posthoc pairwise Wilcoxon and Benjamini-Hochberg corrections were used to compare more than 2 groups.

b) Microfluidics behavioral assays: We used two-way ANOVA with Bonferroni corrections to analyze microfluidics behavioral assay data.

Although we obtain chemotaxis indices indicating behavioral preferences of a population of animals via both microfluidics and plate assays, the underlying data from which the indices are derived are distinct between these two types of assays. Plate chemotaxis assays are end-point assays generating binary data at the 1 hr end point of the assay, whereas the microfluidics chemotaxis index is derived from continuous distributional data of animal position over the entire duration of the assay (20 minutes). Each data point for the microfluidics behavioral assay represents distributional data from 20 worms. Since the underlying data from plate and microfluidics behavioral assays are different, we used different statistical tests to analyze these data sets.

We have now added this information to the Methods section.

*7. For ease of comparison among the many calcium imaging experiments in Figures 2 and 4 where the panels have similar sets of data in a similar order, I suggest:*

*(i) aligning them in portrait orientation; and*

*(ii) using consistent labelling by including the name of the neuron in all Y-axes labels and* *removing the odorant from the Y-axes since they are redundant with the stimulus label.*

Thank you for these suggestions. We have now re-organized Figure 2 as suggested and ensured that X and Y axes labels are consistent across all figures. As suggested by the reviewer, we now include the neuron name on the Y axis labels and have removed the odorant label. We elected to not re-organize Figure 4 similarly since we wished to keep data associated with each genotype in separate rows.

Reviewer #2:

*1. The GPCR odorant receptors that detect the odorants involved in this study are yet to be identified. In the absence of this information the authors are unabvle to test the mechanism of response reversal adequately. Could it be competitive or allosteric interactions at the same receptor? Or some form of integrative effect downstream of two different receptors for the 2 odorants? Is there a receptor specific adaptation or modification? Without the ability to do such experiments the analyses of the phenomenon using downstream factors seems a bit preliminary.*

We are very interested in following up further on this work to characterize the underlying mechanisms including identification and characterization of the relevant receptors. However, we note that this is a major undertaking. The *C. elegans* genome encodes ~1300 chemoreceptors of which only a handful have been de-orphanized to date. Moreover, unlike the general one neuron-one receptor rule in vertebrate and most fly olfactory neurons, each *C. elegans* chemosensory neuron expresses multiple putative chemoreceptor genes. According to the recently published database of single neuron transcriptomes (Taylor et al., 2021), the AWC neuron pair alone is predicted to express ~100 chemoreceptor GPCRs. Thus, attempting to identify which one(s) of these encodes the hexanol and/or the IAA receptor(s) is non-trivial.

Characterizing possible biochemical mechanisms that may operate at the receptor level or downstream to result in the observed response plasticity will further require successful heterologous expression of these receptors. As was originally the case for vertebrate olfactory receptors, heterologous expression of *C. elegans* chemoreceptors is also non-trivial to achieve. We performed this successfully previously (Kim et al., 2009), but this was a laborious and challenging undertaking.

We appreciate the reviewer’s interest in the work but hope that the reviewer agrees that completing these experiments is well beyond the scope for an initial Short Report describing a novel mechanism underlying olfactory behavioral plasticity.

*2. The ecological role of such a phenomenon is unclear and not tested by the authors. What could be the benefit of switching from inhibition (attraction) to excitation (aversion) for the one* *odorant in the presence of IAA is unclear?*

We have now added a paragraph to the Discussion to specifically address this issue:

“Odorants such as IAA and benzaldehyde are derived from amino acid degradation pathways in bacterial species that are preferred attractive food sources for *C. elegans* (Choi et al., 2016; Hazelwood et al., 2008; Tracey and Britz, 1989; Worthy et al., 2018). In contrast, hexanol is produced via chain elongation in bacteria that are likely to be poor food sources and/or pathogenic for *C. elegans* (Thauer et al., 1968). We speculate that *C. elegans* may be attracted to hexanol-producing bacteria under conditions where these bacteria are the only available food source. However, in the presence of odors indicating the presence of more nutritious bacteria, it may be advantageous for worms to instead avoid hexanol. The hexanol-evoked response inversion in AWC may be particularly critical to drive adaptive food choice behavior in the presence of other attractive odorants that are primarily sensed by AWC as compared to chemicals such as diacetyl and pyrazine, attraction to which is largely driven by other olfactory neuron types (Bargmann et al., 1993).”

*3. The authors report the Delta F/F values in the plots showing the activities of AWC, however we get little sense for whether the baseline levels of Ca in the AWC neurons are dramatically different or not. Also, do these neurons fire action potentials like the AWA and if so, it would be useful to also find out the changes in the baseline and stimulus dependent firing rate?*

Please see response to Reviewer 1, points #1 and 2, and new Figures S2C and S5C where we now show raw traces of the fluorescence changes in response to hexanol in control and sIAA conditions in wild-type, *odr-3* and *odr-1* mutants. Please also see Figure R1 above for F0 values in wild-type, *odr-1* and *odr-3* animals. AWC is not known or reported to fire action potentials.

*4. Since this is a unusual phenomenon, understanding it in more detail, such as across multiple doses of the sIAA and hexanol, or across a larger panel of odorants, would have been useful. For example, based on the few chemical structures tested it would appear that the two classes of odorants use distinct binding sites or distinct reporters. Would that interpretation be supported from additional odorants tested?*

To address this comment, we have now performed several additional experiments and fully re-organized and rewritten this section.

a) We specifically selected a sIAA concentration at which a pulse of 10^-4^ IAA would fail to elicit an additional behavioral or calcium response (see Figures S1C and S2B), indicating that the IAA response is saturated. The goal was to also assess the response to 10^-4^ hexanol at this sIAA concentration. To address the reviewer’s concern, we maintained the sIAA concentration at 10^-4^ but varied the hexanol concentration. We find that similar to the responses observed using 10^-4^ hexanol, 10^-3^ and 10^-5^ hexanol also decreased intracellular calcium in AWC in control conditions, but increased it in sIAA (new Figure S2D).

b) We had previously tested several odorant/saturating odorant combinations for responses in AWC. We have now added to these data as requested by the reviewer.

i) To test the extent to which sIAA inverts the response to other AWC-sensed odorants, we had previously examined responses to a pulse of IAA, hexanol, heptanol, benzaldehyde, and pyrazine. We have now added new data for diacetyl (new Figure S3A); we find that a pulse of diacetyl does not activate AWC in sIAA. We conclude that in sIAA, the observed inversion of the response sign in AWC is largely specific to hexanol and heptanol.

ii) To test the extent to which saturating with AWC-sensed chemicals other than IAA inverts hexanol responses, we had previously tested hexanol responses in saturating benzaldehyde. We have now added new data for hexanol responses in saturating pyrazine and diacetyl (new Figures S3D and S3E). Interestingly, we observe that saturation with either diacetyl or pyrazine does not invert the hexanol-evoked response in AWC, indicating that this response is restricted to saturation of AWC by only a subset of AWC-sensed odorants.

All these data are now summarized in new Figure S3F, and implications of these data are addressed in the Discussion.

Reviewer #3:

*1. However, there are some issues that somewhat reduce my enthusiasm. Most important of these is that the paper concludes without identifying the mechanism by which sIAA alters AWC physiology to reconfigure HEX/HEPT reponses. As a Short Report, this might not be a concern, but I'm sure whether that the level of general interest here is high enough to compensate for that.*

As we hope the Reviewer appreciates, we report a novel mechanism of olfactory sensory plasticity. We have identified a subset of the underlying mechanisms and hope to be able to identify and characterize additional mechanisms going forward. As described further below, we have also now characterized responses of *daf-11* mutants and have thus further characterized the intracellular signaling pathway.

*2a. One possibility that seems worth exploring more deeply would be the role of cGMP. Under sIAA conditions, [cGMP] in AWC might be quite low - I wonder whether artificially lowering [cGMP] under control conditions might cause HEX/HEPT to become repellants.*

We appreciate the reviewer’s comment but think that this is unlikely to be the case. Under control conditions, intracellular [cGMP] is largely generated by the ODR-1 and DAF-11 rGCs (see Figure 4C and new Figures S5D-E). Thus, in *odr-1* and *daf-11* mutants, intracellular [cGMP] is expected to be low. In fact, addition of odorants has previously been shown to fail to further alter intracellular [cGMP] in AWC in *odr-1* mutants (Shidara et al., 2017). Consistently, as shown in Figure 4C and new Figures S5D-E, under control conditions in *odr-1* and *daf-11* mutants, hexanol responses are absent in AWC, suggesting that simply abolishing cGMP production in control conditions is unlikely to be sufficient to trigger an increased calcium response and drive avoidance. Besides examining rGC mutants (please also see response to point 2b below), we are not able to currently artificially lower [cGMP] in AWC *in vivo*.

More importantly, the hexanol-evoked intracellular calcium increase in sIAA requires the cGMP-gated channels TAX-2/4 (Figure 4A), indicating that cGMP levels must rise upon hexanol addition in sIAA to open these channels and activate AWC.

*2b. A related issue is about the role of the RGC odr-1 - it's fascinating that loss of odr-1 nearly recapitulates the effect of sIAA with respect to HEX behavior and pushes the Ca++ response in the same direction. Maybe the Ca++ response to HEX would invert (and generate stronger HEX repulsion) in daf-11 or daf-11; odr-1 animals? I wonder whether somehow increasing [cGMP] in AWC might suppress the odr-1 HEX-aversion phenotype (and/or the effect of sIAA). Maybe odr-1 mutants are defective for IAA attraction because the normal IAA signaling pathway is already being pushed towards "saturation"?*

We may be misinterpreting the reviewer’s comment but is the reviewer referring to the avoidance observed in *odr-1* mutants in control conditions as ‘nearly recapitulating’ the avoidance behavior of WT animals in sIAA (Figure 4B)? While the avoidance behaviors (WT in sIAA and *odr-1* in control) are indeed similar, these responses are mediated via distinct mechanisms.

In WT in sIAA, avoidance is driven by the activation of AWC as well as by ASH. The evidence for this is provided in Figures 1D and 1E which shows that ablation of both AWC and ASH is necessary to abolish avoidance in sIAA.

In *odr-1* in control conditions, avoidance is driven only by ASH. The AWC neurons are unable to respond to hexanol in *odr-1* mutants in control conditions (Figure 4C) and thus, AWC-mediated attraction is no longer able to antagonize ASH-driven avoidance. As a consequence, in *odr-1* mutants (or upon AWC ablation, Figures 1D and 1E), ASH-driven avoidance predominates.

The mechanistic difference underlying the avoidance behavior of WT in sIAA and *odr-1* in control conditions is specifically manifested in the hexanol-evoked calcium responses in AWC. As shown in Figure 4C, hexanol activates AWC in sIAA in wild-type animals. In *odr-1* mutants in control conditions, hexanol fails to inhibit AWC.

However, the reviewer raises an interesting point regarding a possible role for DAF-11 in the hexanol response. To address this, we have now examined hexanol-evoked responses in AWC in *daf-11(m47)* mutants. As shown in new Figure S5D, we find that *daf-11* mutants exhibit no responses to a pulse of hexanol under control conditions similar to our observations in *odr-1* mutants. In sIAA, hexanol again activates AWC in *daf-11* mutants albeit at a lower frequency than in wild-type or *odr-1* mutants.

We conclude that both ODR-1 and DAF-11 are necessary for hexanol-mediated inhibition of AWC in control conditions. However, while DAF-11 may be partly necessary for driving hexanol-evoked activation of AWC in sIAA, additional DAF-11- and ODR-1-independent pathways likely contribute to hexanol-evoked activation of AWC under these conditions. We were unable to examine *daf-11; odr-1* double mutants since these animals constitutively enter the dauer stage. AWC is predicted to express at least six receptor guanylyl cyclases including ODR-1 and DAF-11, as well as five phosphodiesterases (Taylor et al., 2021). ODR-3 may activate DAF-11 and additional guanylyl cyclases other than ODR-1, and/or inhibit one or more phospodiesterases to increase intracellular cGMP levels in sIAA (revised Figure 4D).

To attempt to examine the reviewer’s suggestion of examining the effects of increasing intracellular [cGMP] on hexanol responses in wild-type and *odr-1* mutants, we assayed the behaviors of wild-type and *odr-1* mutants grown on plates containing the non-hydrolyzable cGMP analog 8-Br-cGMP. We used a concentration of this chemical that we previously demonstrated to have physiological effects on behavior and neuronal morphology (Mukhopadhyay et al., 2008; Wasserman et al., 2011). However, as shown in Figure R2 below, we observed no changes in the behaviors of wild-type or *odr-1* mutants upon growth on this chemical.

**Fig. R2**. Average residency plot histograms in microfluidics behavioral devices with a central stripe of 10^-4^ hexanol without (left), or with, a uniform concentration of 10^-4^ IAA (right) as described in Fig 1C-D. WT and *odr-1(n1936)* animals were cultivated for one generation without or with 5mM 8-Br-cGMP. Mean relative residence >1 or <1 (dashed vertical lines) indicate attraction and avoidance, respectively. n=20-30 animals per assay; 3 biological replicates.

*9. Is there any reason to believe that worms might encounter persistent, saturating concentrations of AWC-sensed odorants in the wild? The idea that this mechanism could help animals distinguish between food sources is interesting, but it also seems possible (even likely) that that the behavioral inversion seen in the lab is the result of pushing the system beyond its normal dynamic range. That doesn't make these findings uninteresting - they tell us something important about how AWC works - but they limit the potential that the phenomenon identified here has any* adaptive value.

See response to Reviewer 2, point #2.

*10. Fig 1C-E - please indicate that the odorant is HEX.*

Done.

*11. Fig S2B clearly shows that AWC can still somehow see changes in IAA concentration in sIAA. What's the significance of this? How can it be reconciled with the lack of IAA attraction in sIAA?*

As shown in the quantification in Figure S2B, addition of IAA results in a very small decrease in intracellular calcium in sIAA as compared to the large amplitude of the decrease in control conditions. The amplitude of the OFF response upon removal of IAA is also smaller in sIAA than in control conditions. These small changes in intracellular calcium are not sufficient to drive behavioral changes under the assay conditions shown in Figure 1A.

*12. The response of AWC to 2-methylpyrazine (Fig S2E) seems surprising given, as the authors note, that this compound is thought to be detected primarily by AWA. It would be useful for the authors to comment on the potential significance of this result.*

As the reviewer is likely aware, odorants are detected by different subsets of sensory neurons in *C. elegans* at different concentrations and under different conditions. As far as we are aware, there is little work systematically examining pyrazine responses via calcium imaging in *C. elegans* sensory neurons. We were also surprised to observe this response. The role of this response in AWC in driving behavior is currently unclear, and we hope that the reviewer agrees that further speculation is tangential to the main focus of this work.

*13. Fig 3C: Please add an "odorant HEX" label*

Done.

*14. p. 13: "A trivial explanation for the observed phenotype is that ODR-3 may be required for IAA-mediated saturation in AWC, leading to similar hexanol-evoked responses in odr-3 mutants in both unsaturated and saturated conditions." This may be a straightforward possibility, but I don't think it's a trivial one. It would still provide important insight into the diversity of signaling mechanisms in AWC.*

The reviewer’s point is well-taken. We have replaced ‘trivial’ with ‘possible’.

*15. p. 13: Please add "...in odr-3 mutants" to "Consistent with previous work showing that AWC retains partial ability to respond to IAA"*

Done.

*In summary*, in response to Reviewers’ comments, we made extensive revisions to the text and Figures and also added new experimental data and analyses. New data and analyses are now presented in Figures S2C, S2D, S3A, S3D, S3E, S3F, 4D, S5C, S5D, S5E, R1 and R2 (R1 and R2 are in this document). We thank the reviewers for their comments and their interest and hope that they find the revisions satisfactory. We look forward to following up on this work further in the future.

**REFERENCES**

Albrecht, D.R., and Bargmann, C.I. (2011). High-content behavioral analysis of *Caenorhabditis elegans* in precise spatiotemporal chemical environments. Nat Methods *8*, 599-605.

Bargmann, C.I., Hartwieg, E., and Horvitz, H.R. (1993). Odorant-selective genes and neurons mediate olfaction in *C. elegans*. Cell *74*, 515-527.

Birnby, D.A., Link, E.A., Vowels, J.J., Tian, H., Colacurcio, P.L., and Thomas, J.H. (2000). A transmembrane guanylyl cyclase (DAF-11) and Hsp90 (DAF-21) regulate a common set of chemosensory behaviors in *C. elegans*. Genetics *15,* 85-104.

Chalasani, S.H., Kato, S., Albrecht, D.R., Nakagawa, T., Abbott, L.F., and Bargmann, C.I. (2010). Neuropeptide feedback modifies odor-evoked dynamics in *Caenorhabditis elegans* olfactory neurons. Nat Neurosci *13*, 615-621.

Cho, C.E., Brueggemann, C., L'Etoile, N.D., and Bargmann, C.I. (2016). Parallel encoding of sensory history and behavioral preference during *Caenorhabditis elegans* olfactory learning. Elife *5*, e14000.

Choi, J.I., Yoon, K.H., Subbammal Kalichamy, S., Yoon, S.S., and Il Lee, J. (2016). A natural odor attraction between lactic acid bacteria and the nematode *Caenorhabditis elegans*. ISME J *10*, 558-567.

Hazelwood, L.A., Daran, J.M., van Maris, A.J., Pronk, J.T., and Dickinson, J.R. (2008). The Ehrlich pathway for fusel alcohol production: a century of research on *Saccharomyces cerevisiae* metabolism. Appl Environ Microbiol *74*, 2259-2266.

Kim, K., Sato, K., Shibuya, M., Zeiger, D.M., Butcher, R.A., Ragains, J.R., Clardy, J., Touhara, K., and Sengupta, P. (2009). Two chemoreceptors mediate developmental effects of dauer pheromone in *C. elegans*. Science *326*, 994-998.

Kunitomo, H., Sato, H., Iwata, R., Satoh, Y., Ohno, H., Yamada, K., and Iino, Y. (2013). Concentration memory-dependent synaptic plasticity of a taste circuit regulates salt concentration chemotaxis in *Caenorhabditis elegans*. Nat Commun *4*, 2210.

Larsch, J., Flavell, S.W., Liu, Q., Gordus, A., Albrecht, D.R., and Bargmann, C.I. (2015). A circuit for gradient climbing in *C. elegans* chemotaxis. Cell Rep *12*, 1748-1760.

Levy, S., and Bargmann, C.I. (2020). An adaptive-threshold mechanism for odor sensation and animal navigation. Neuron *105*, 534-548 e513.

Luo, L., Wen, Q., Ren, J., Hendricks, M., Gershow, M., Qin, Y., Greenwood, J., Soucy, E.R., Klein, M., Smith-Parker, H.K.*, et al.* (2014). Dynamic encoding of perception, memory, and movement in a *C. elegans* chemotaxis circuit. Neuron *82*, 1115-1128.

Mukhopadhyay, S., Lu, Y., Shaham, S., and Sengupta, P. (2008). Sensory signaling-dependent remodeling of olfactory cilia architecture in *C. elegans*. Dev Cell *14*, 762-774.

Pierce-Shimomura, J.T., Morse, T.M., and Lockery, S.R. (1999). The fundamental role of pirouettes in *Caenorhabditis elegans* chemotaxis. J Neurosci *19*, 9557-9569.

Shidara, H., Hotta, K., and Oka, K. (2017). Compartmentalized cGMP responses of olfactory sensory neurons in *Caenorhabditis elegans*. J Neurosci *37*, 3753-3763.

Sieburth, D., Madison, J.M., and Kaplan, J.M. (2007). PKC-1 regulates secretion of neuropeptides. Nat Neurosci *10*, 49-57.

Speese, S., Petrie, M., Schuske, K., Ailion, M., Ann, K., Iwasaki, K., Jorgensen, E.M., and Martin, T.F. (2007). UNC-31 (CAPS) is required for dense-core vesicle but not synaptic vesicle exocytosis in *Caenorhabditis elegans*. J Neurosci *27*, 6150-6162.

Taylor, S.R., Santpere, G., Weinreb, A., Barrett, A., Reilly, M.B., Xu, C., Varol, E., Oikonomou, P., Glenwinkel, L., McWhirter, R.*, et al.* (2021). Molecular topography of an entire nervous system. Cell *184*, 4329-4347 e4323.

Thauer, R.K., Jungermann, K., Henninger, H., Wenning, J., and Decker, K. (1968). The energy metabolism of *Clostridium kluyveri*. Eur J Biochem *4*, 173-180.

Tracey, R.P., and Britz, T.J. (1989). Freon 11 extraction of volatile metabolites formed by certain lactic acid bacteria. Appl Environ Microbiol *55*, 1617-1623.

Ventimiglia, D., and Bargmann, C.I. (2017). Diverse modes of synaptic signaling, regulation, and plasticity distinguish two classes of *C. elegans* glutamatergic neurons. Elife *6*, e31234.

Wasserman, S.M., Beverly, M., Bell, H.W., and Sengupta, P. (2011). Regulation of response properties and operating range of the AFD thermosensory neurons by cGMP signaling. Curr Biol *21*, 353-362.

Worthy, S.E., Haynes, L., Chambers, M., Bethune, D., Kan, E., Chung, K., Ota, R., Taylor, C.J., and Glater, E.E. (2018). Identification of attractive odorants released by preferred bacterial food found in the natural habitats of *C. elegans*. PLoS One *13*, e0201158.
